# ThNAC13, a NAC Transcription Factor from *Tamarix hispida*, Confers Salt and Osmotic Stress Tolerance to Transgenic *Tamarix* and *Arabidopsis*

**DOI:** 10.3389/fpls.2017.00635

**Published:** 2017-04-26

**Authors:** Liuqiang Wang, Zhen Li, Mengzhu Lu, Yucheng Wang

**Affiliations:** ^1^State Key Laboratory of Tree Genetics and Breeding, Key Laboratory of Tree Breeding and Cultivation of the State Forestry Administration, Research Institute of Forestry, Chinese Academy of ForestryBeijing, China; ^2^Key Laboratory of Biogeography and Bioresource in Arid Land, Xinjiang Institute of Ecology and Geography, Chinese Academy of SciencesUrumqi, China

**Keywords:** abiotic stress, NAC transcription factor, ROS-scavenging, stress resistance, *Tamarix hispida*

## Abstract

NAC (NAM, ATAF1/2, and CUC2) proteins play critical roles in many plant biological processes and environmental stress. However, NAC proteins from *Tamarix hispida* have not been functionally characterized. Here, we studied a *NAC* gene from *T*. *hispida, ThNAC13*, in response to salt and osmotic stresses. ThNAC13 is a nuclear protein with a C-terminal transactivation domain. ThNAC13 can bind to NAC recognized sites and calmodulin-binding NAC (CBNAC) binding element. Overexpression of *ThNAC13* in *Arabidopsis* improved seed germination rate and increased root growth and fresh weight gain under salt or osmotic stress. Transgenic *T*. *hispida* plants transiently overexpressing *ThNAC13* and with RNAi-silenced *ThNAC13* were generated for gain- and loss-of-function experiments. Following exposure to salt or osmotic stress, overexpression of *ThNAC13* induced superoxide dismutase (SOD) and peroxidase (POD) activities, chlorophyll and proline contents; decreased the reactive oxygen species (ROS) and malondialdehyde levels; and reduced electrolyte leakage rates in both transgenic *Tamarix* and *Arabidopsis* plants. In contrast, RNAi-silenced *ThNAC13* showed the opposite results in transgenic *Tamarix*. Furthermore, *ThNAC13* induced the expression of *SOD*s and *POD*s in transgenic *Arabidopsis*. These results suggest that ThNAC13 improves salt and osmotic tolerance by enhancing the ROS-scavenging capability and adjusting osmotic potential.

## Introduction

Transcriptional regulation plays crucial roles in controlling plant biological processes and signaling pathways, and transcription factors specifically interact with promoter elements of target genes to activate or repress their transcription (Pérez-Rodríguez et al., 2009; Shang et al., 2013). In plants, several families of stress-responsive transcription factors have been functionally characterized in stress regulation networks, such as NAC, bZIP, WRKY, MYB/MYC and AP2/ERF (Le Hénanff et al., 2013; Shiriga et al., 2014).

The NAC (NAM, ATAF1/2, and CUC2) transcription factors are plant-specific proteins, which comprise one of the largest transcription factors families, and are involved in various plant biological processes, such as lateral root formation, cell division, wood formation and xylary fiber development (Zhong et al., 2010; Hao et al., 2011; Lin et al., 2013; Zhao et al., 2014) and environmental stress responses (Nakashima et al., 2012; Shao et al., 2015; He et al., 2017). Most NAC proteins contain a highly conserved N-terminus domain and a diversified C-terminal domain (Puranik et al., 2012). To date, some *NAC* genes have been shown to be involved in abiotic stress. In *Arabidopsis* and rice, the stress-responsive *NAC* gene, *ANAC096*, was reported to have a synergistic relationship with ABRE binding factor and increased the plant survival rate under osmotic and drought stresses (Xu et al., 2013). AtNAC019, AtNAC055, and AtNAC072 could specifically bind to the NAC recognized site (NACRS) in the promoter of the *EARLY RESPONSIVE TO DEHYDRATION STRESS 1* (*ERD1*) gene to enhance drought tolerance (Tran et al., 2004; Hickman et al., 2013). The *Arabidopsis NAC016* can repress the transcripts of *ABSCISIC ACID-RESPONSIVE ELEMENT BINDING PROTEIN 1* (*AREB1*), which is a key transcription factor in the ABA-dependent stress-signaling pathway, and then promote responses to drought stress (Sakuraba et al., 2015). *OsNAC5* expression is induced by drought, high salinity and ABA, enhancing tolerance to drought, salinity and low temperature in transgenic rice (Jeong et al., 2013). Hong et al. (2016) demonstrated that *ONAC022* expression was a response to abiotic stress, and transgenic rice overexpressing *ONAC022* showed enhanced tolerance to drought and salt stresses by modulating the ABA signaling pathway. In addition, many NAC proteins have been shown to play important roles in modulating ROS levels. For example, Fang et al. (2015) reported that *SNAC3* was ubiquitously expressed under drought, salinity and ABA treatments. Overexpression of *SNAC3* in transgenic rice conferred tolerance to high temperature, drought and oxidative stresses through modulation of ROS scavenging pathways. Oilseed rape *NAC56* and *NAC55* could induce the expression of genes involved in ROS scavenging to decrease ROS accumulation, leading to improved abiotic stress tolerance (Niu et al., 2016; Chen et al., 2017). In contrast, sweet potato *IbNAC1* could increase ROS in response to jasmonic acid and plays a role in reprogramming the transcriptional response to wounding *via* the JA-mediated pathway (Chen et al., 2016). In soybean, *GmNAC20* and *GmNAC11* are involved in different abiotic stresses, and their expression in *Arabidopsis* conferred both salt and freezing tolerance (Hao et al., 2011). Overexpression of horsegram *MuNAC4* in transgenic groundnut plants reduces the damage to membrane structures and enhances osmotic adjustment and antioxidative enzyme regulation under drought stress (Pandurangaiah et al., 2014). *TaNAC29* and *TaNAC67* are up-regulated under different abiotic stresses, and transgenic *Arabidopsis* plants expressing these genes showed increased salt and drought tolerance (Mao et al., 2014; Huang et al., 2015). In addition, a NAC-type transcription factor from chickpea, CarNAC4, induces the expression of genes involved in stress tolerance, including *DREB2A, COR15A, RD29A*, and *KIN1*, which increased tolerance to salt and drought stresses (Yu et al., 2016).

Although different NAC transcription factors have been successively identified in several plant species and reported to be involved in abiotic stress, the specific biological functions of NAC transcription factors from halophyte woody plants have not been fully elucidated. *Tamarix* (Tamaricaceae) is a family of woody halophyte species, which includes small trees or shrubs, and is extensively distributed in saline soils of drought-stricken areas of Central Asia and China. *Tamarix hispida*, a species of the genus *Tamarix*, is highly tolerant to salinity, drought and extreme temperatures. These characteristics make this species a suitable plant for investigation of the mechanism of stress tolerance. We previously identified 21 full-length coding region *NAC* genes from *T*. *hispida* and found that *ThNAC13* was predominantly up-regulated by salt and drought treatments (Wang et al., 2014b). In this study, we performed a detailed functional analysis of *ThNAC13* in response to salt and osmotic stresses. ThNAC13 protein is localized in the cell nucleus and has transactivation activity at the C-terminus. *T*. *hispida* plants transiently overexpressing and with RNAi-silencing of *ThNAC13* were generated to investigate the tolerance to salt and osmotic stresses. Furthermore, *Arabidopsis* plants overexpressing *ThNAC13* were also generated and used to further confirm the results obtained from *T. hispida* plants. Our results suggest that *ThNAC13* is a potential candidate for enhancing tolerance to salt and osmotic stresses *via* genetic breeding.

## Materials and Methods

### Plant Materials and Growth Conditions

*Tamarix hispida* plants were used for gene cloning, transgenic analysis and expression analysis. The seedlings of *T. hispida* were cultured in tissue culture bottles containing half-strength Murashige-Skoog (1/2 MS) solid medium [2% (w/v) agar] in a culture room at average temperature of 24°C with 14 h light/10 h darkness photocycle conditions. *Arabidopsis thaliana* (ecotype Columbia) was used for genetic transformation and functional analysis. Seven-day-old seedlings were grown in pots filled with soil and perlite in a controlled chamber (16 h light/8 h darkness photocycle; 70–75% relative humidity; 22°C).

### Isolation and Bioinformatic Analysis of *ThNAC13*

The cDNA sequence of *ThNAC13* (GenBank number: JQ974967) was identified from the transcriptomes of *T. hispida* (Wang et al., 2014b). Multiple sequence alignments of the deduced protein sequence of ThNAC13 together with other reported stress-responsive NACs from different plant species were deduced using ClustalW software, and the phylogenetic tree was constructed with MEGA (version 5.1) program (Tamura et al., 2011). The nuclear localization signal of ThNAC13 was predicted using the WoLFPSORT algorithm (Horton et al., 2007). The conserved motifs among the full length NAC protein sequences were predicted using MEME online program^1^ (Bailey et al., 2009).

### Subcellular Localization Analysis of ThNAC13 Protein

The full-length coding region of *ThNAC13* (without stop codon) was cloned into the pBI121 vector containing the *GFP* reporter gene driven by the CaMV 35S promoter to generate the 35S:ThNAC13-GFP construct. The 35S:ThNAC13-GFP and the 35S:GFP (control) constructs were separately introduced into live onion epidermal cells using the particle bombardment method and visualized by a confocal laser scanning microscope (Wang et al., 2014a). The nuclei were stained with 4,6′-diamidino-2-phenylindole (DAPI, 10 μg.mL^-1^) in phosphate-buffered saline for 3 min and analyzed.

### Transcriptional Activation Assay

To study the transactivation activity of ThNAC13, the full coding region and a series of truncated *ThNAC13* genes were cloned into the pGBKT7 vector (Clontech, USA) to fuse with a *GAL4* DNA binding domain. Fusion plasmids and the empty pGBKT7 vector (negative control) were individually transformed into the yeast strain Y2HGold and grown on SD medium lacking tryptophan (Trp; SD/-Trp) supplemented with X-α-Gal (5-bromo-4-chloro-3-indolyl-α-D-galactopyranoside) for 2–3 days of culture at 30°C. Yeast transformation and blue/white colony assays were performed following to the manufacturer’s instructions (Clontech, USA). The transcriptional activation activities were evaluated according to the growth status of the transformants.

### Yeast One-Hybrid (Y1H) Assay

Two tandem copies of the NACRS with the core sequence “CGT[A/G]” and calmodulin-binding NAC (CBNAC) binding site with the core sequence “GCTT” were separately inserted into the pHIS2 vector (Clontech, USA) as the reporter constructs. The coding region of *ThNAC13* gene was cloned into the pGADT7-Rec2 vector as the effector construct. The effector construct was co-transformed together with each reporter construct into the yeast strain Y187. The DNA-protein interactions were evaluated according to the growth ability of the cotransformants on SD medium lacking leucine (Leu) and tryptophan (Trp; SD/-Leu/-Trp, DDO) and SD medium without leucine (Leu), tryptophan (Trp) and histidine (His; SD/-Leu/-Trp/-His, TDO) containing 3-AT (3-amino-1,2,4-triazole).

### Generation of Transgenic Plants

The full coding sequence (CDS) of *ThNAC13* was cloned into pROKII under the control of the CaMV 35S promoter (35S:ThNAC13) and transformed into *A*. *thaliana* according to the protocol described by Clough and Bent (1998). The positive transgenic lines were selected on kanamycin (50 mg/L) plates, and further identified by genomic DNA PCR, and the ThNAC13 expression level in leaves of each transgenic line was examined by quantitative reverse transcription polymerase chain reaction (qRT-PCR). The homozygous lines of T_3_ generation plants were used for study.

The sense and antisense sequences of the specific coding region of *ThNAC13* with 259 bp in length were cloned into the RNAi vector (pFGC5941) to generate pFGC:ThNAC13 and silence the expression of *ThNAC13*. Transient transformation of 6-week-old *T. hispida* plants was performed according to Ji et al. (2014) with some modifications. Briefly, the whole seedlings were soaked in the transformation solution [1/2 MS + 150 μM acetosyringone + 3% (w/v) sucrose + 0.01% (w/v) Tween-20, pH 5.8] with *Agrobacterium tumefaciens* EHA105 strain at 0.6 OD_600_ and incubated with shaking at 100 r/min for 4 h at 25°C. Then, the seedlings were washed with distilled water twice and whipped with sterile facial tissue to remove excessive water. The whole seedlings were grown vertically on 1/2 MS agar medium [150 μM acetosyringone + 2.5% (w/v) sucrose, pH 5.8] in tissue culture bottles. Three types of transient transgenic *T*. *hispida* plants were generated: *T*. *hispida* overexpressing *ThNAC13* (transformed with 35S:ThNAC13, OE), *T*. *hispida* RNAi-silencing *ThNAC13* (transformed with pFGC:ThNAC13, IE) and plants transformed with empty pROKII vector as a control (VC). The expression of *ThNAC13* in the leaves of these transformed *T*. *hispida* plants was studied using qRT-PCR.

### Analysis of Stress Tolerance

Two representative transgenic lines overexpressing *ThNAC13* and wild type (WT) *Arabidopsis* plants were selected for abiotic stress tolerance assays. For germination rate assays, the seeds were surface-sterilized and sowed on 1/2 MS solid medium supplemented with 100 mM NaCl, 200 mM mannitol or 2 μM ABA and incubated at 22°C. The rates of seeds were calculated to determine when the seedling opened green cotyledons. Next, 5-day-old seedlings were cultured vertically on 1/2 MS solid medium supplemented with 100 mM NaCl, 200 mM mannitol or 10 μM ABA. After 7 days of these treatments, the fresh weight and the length of primary roots of each plant were measured.

### NBT and DAB Staining

NBT and DAB *in situ* staining for detection of hydrogen peroxide (H_2_O_2_) and superoxide ($O_{2}^{•–}$) were performed. The transformed seedlings of *T. hispida* were exposed to 150 mM NaCl, 200 mM mannitol or 30 μM ABA for 24 h. Four-week-old soil-grown *Arabidopsis* plants were treated with the same concentration of NaCl, mannitol or ABA for 2 h, and approximately 10 young branches harvested from *T*. *hispida* and 10 rosette leaves harvested from *Arabidopsis* were incubated with nitroblue tetrazolium (NBT) or 3,3′-diaminobenzidine (DAB) solutions for staining (Zhang et al., 2011).

### Measurement of Physiological Changes

The transformed *T*. *hispida* plants grown on 1/2 MS solid medium supplemented with 150 mM NaCl, 200 mM mannitol or 30 μM ABA for 24 h were used to assess physiological changes. Four-week-old soil-grown *Arabidopsis* seedlings were treated with same concentration of NaCl, mannitol or ABA for 2 h (for H_2_O_2_ content measurement) and 48 h (for other physiological analyses). H_2_O_2_ levels were determined according to Thordal-Christensen et al. (1997). Malondialdehyde (MDA) contents, SOD and POD activities were measured according to Wang et al. (2010). Electrolyte leakage was determined following the protocol described by Ben-Amor et al. (1999). Proline contents were measured according to Bates et al. (1973). Relative chlorophyll contents were calculated using a chlorophyll analyzer (Konica Minolta, Japan). At least 20 seedlings were employed for physiological indices analysis in each sample.

### Real-Time Quantitative RT-PCR Analysis

Total RNA was isolated from leaves of *T*. *hispida* and *Arabidopsis* plants using a CTAB (hexadecyltrimethylammonium bromide) method (Chang et al., 1993). The first-strand cDNA was synthesized using the PrimeScript^TM^ RT reagent Kit (TaKaRa, China), and the real-time RT-PCR was performed in the Opticon 2 System (Bio-Rad, Hercules, CA, USA) following the protocol described by Wang et al. (2014b). The relative expression levels were determined using the 2^-ΔΔCT^ method (Livak and Schmittgen, 2001). All primer sequences used in this study are shown in Supplementary Table S1.

### Statistical Analyses

Each experiment was repeated independently at least three times. The error bars represent standard deviations. Statistical analyses were carried out using Excel software. The data were compared using Student’s *t*-test, and statistical differences were considered to be significant if *P* < 0.05. Asterisks indicates significant difference (^∗^*P* < 0.05).

## Results

### Bioinformatics Analysis of *ThNAC13* from *T. hispida*

**FIGURE 1:**
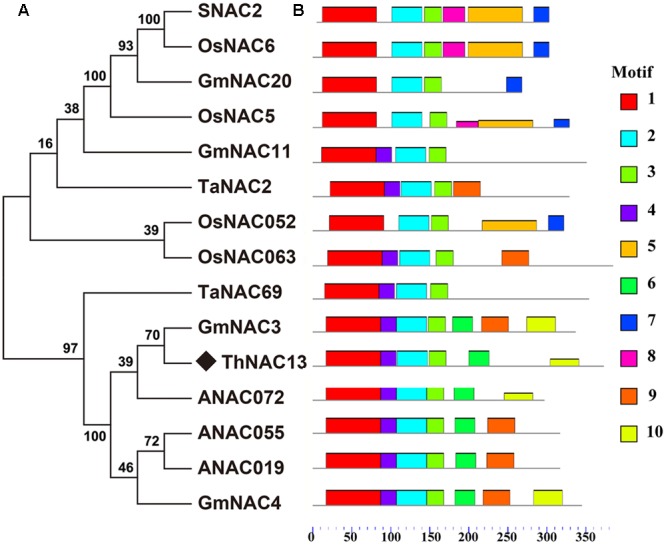
**Bioinformatics analysis of the ThNAC13 protein. (A)** Phylogenetic tree was constructed from ThNAC13 and other different NAC proteins using MEGA 5.1 with 1000 bootstrap replicates. Bootstrap support values are indicated on each node. All protein sequences were retrieved from NCBI. Their corresponding accession numbers are as the follows: *Tamarix hispida* ThNAC13 (JQ974967); *Oryza sativa* SNAC2 (CBX55846), OsNAC5 (BAA89799), OsNAC6 (BAA89800), OsNAC052 (AAT44250), and OsNAC063 (NP_001061889); *Glycine max* GmNAC3 (AAY46123), GmNAC4 (AAY46124), GmNAC11 (ACC66315), and GmNAC20 (ACC66314); *Triticum aestivum* TaNAC2 (AAU08786) and TaNAC69 (AAU08785); *Arabidopsis thaliana* ANAC019 (NP_175697.1), ANAC072 (NP_567773.1), and ANAC055 (NP_188169.1). **(B)** Schematic of putative conserved motifs among these NAC protein sequences was obtained using the MEME program. Different predicted motifs are represented by numbered boxes (annotations of individual motifs are listed in Supplementary Table S2).

The full CDS of *ThNAC13* is 1122 bp in length and encodes a 373 amino acid protein with a molecular weight of 40.74 kDa. Multiple sequence alignments showed that ThNAC13 contains a highly conserved DNA-binding domain at N-terminal, which is a typical NAC domain N-terminal with five subdomains (A, B, C, D, and E), and a highly variable C-terminal transcriptional regulation domain (Supplementary Figure S1). The putative nuclear localization signal of ThNAC13 was identified in the D subdomain. Phylogenetic analysis revealed that ThNAC13 shares high sequence similarities with the plant stress-responsive NAC members (**Figure 1**), indicating that *ThNAC13* may have similar functions to these *NAC*s. Ten conserved motifs (termed motif 1–10) were predicted based on the sequence features of NAC proteins using the MEME program, which are listed in Supplementary Table S2. Motif 1 corresponds to the A and B subdomains, and motifs 2, 3, and 4, respectively, correspond to subdomains C, D, and E.

### Subcellular Location and Transactivation Activity Assay

**FIGURE 2:**
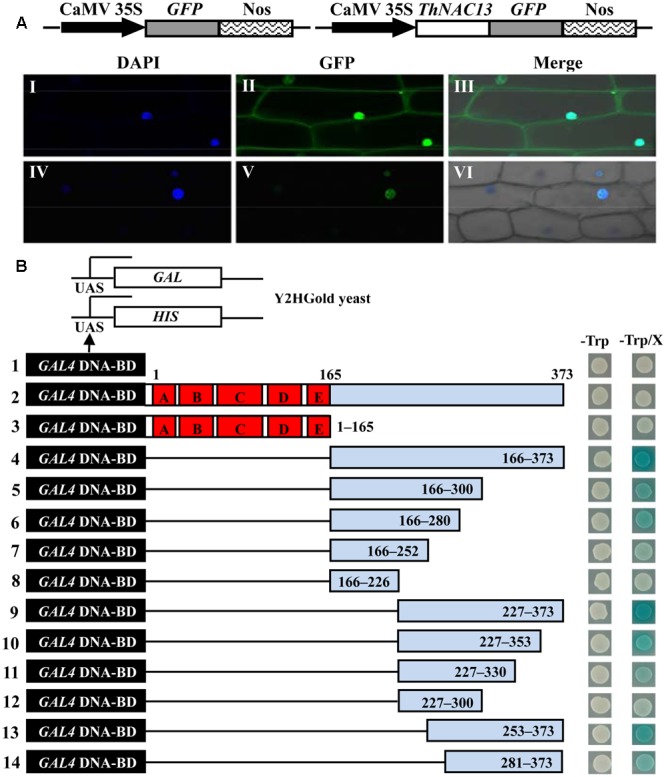
**Nuclear localization and transcriptional activation of the ThNAC13 protein. (A)** Nuclear localization of the ThNAC13 protein. Schematic of the GFP and ThNAC13-GFP fusion constructs. The *GFP* (I–III, control) and *ThNAC13-GFP* (IV–VI) fusion gene were expressed transiently in onion epidermal cells and observed with confocal microscopy. DAPI, DAPI for nuclear staining image; GFP, GFP green fluorescence image; Merge, the merged images of bright-field, GFP and DAPI staining. **(B)** Transcriptional activation activity analysis of ThNAC13 in yeast. Diagram showing a full-length (construct 2) and a series of deletion mutations of the *ThNAC13* gene (constructs 3–14) fused to the downstream of the *GAL4* DNA binding domain in the yeast vector pGBKT7. The pGBKT7 vector (control, constructs 1) and the fusion proteins (constructs 2–14) were expressed in yeast strain Y2HGold. The positive transformants were grown on SD/-Trp medium supplemented with or without X-α-Gal and applied to β-gal assay.

To determine the localization of the ThNAC13 protein, the 35S:GFP (control) and 35S:ThNAC13-GFP constructs were transformed into live onion epidermal cells. The GFP fluorescence of ThNAC13-GFP was exclusively observed in the nucleus, whereas the fluorescence of GFP was distributed in the whole cells (**Figure 2A**). These results clearly indicate that ThNAC13 is a nuclear protein.

To determine the transcriptional activation ability of ThNAC13, the full CDS and serial deletions of the *ThNAC13* CDS were cloned into the pGBKT7 vector (**Figure 2B**, construct 2–14). The different constructs were then transformed into the Y2HGold yeast cells and were assayed for their transcriptional activation. All transformants grew well on the SD/-Trp medium (left panel), indicating that these constructs had been successfully transformed into Y2HGold cells. On the SD/-Trp medium containing X-α-Gal (SD/-Trp/X), the cells harboring full-length CDS of ThNAC13 (construct 2, amino acid 1–373) or empty pGBKT7 (construct 1, control) were not blue (right panel), suggesting that the full CDS of ThNAC13 does not have transcriptional activation ability. However, the yeast cells containing the C-terminal putative activation domain (construct 4, amino acid 166–373) of ThNAC13 grew well and were blue, whereas the N-terminal domain (construct 3, amino acid 1–165) was not blue, suggesting that only the C-terminal has transactivation ability. With the exception of the amino acid 227–373 construct (construct 9), the other deletions of the C-terminal domain exhibited relatively weak transactivation activity. The truncated CDS with amino acids 227–373 still had transactivation activity comparable to that with the whole C-terminus, while construct 8 (amino acid 166–226) completely abolished transactivation activity. These results indicate that ThNAC13 contains the transcriptional activation domain at the region of amino acids 227–373, although the full CDS of *ThNAC13* does not have transcriptional activation, which may be because the N-terminal of ThNAC13 has transcriptional repression activity.

### ThNAC13 Binds to NACRS and CBNACBS

**FIGURE 3:**
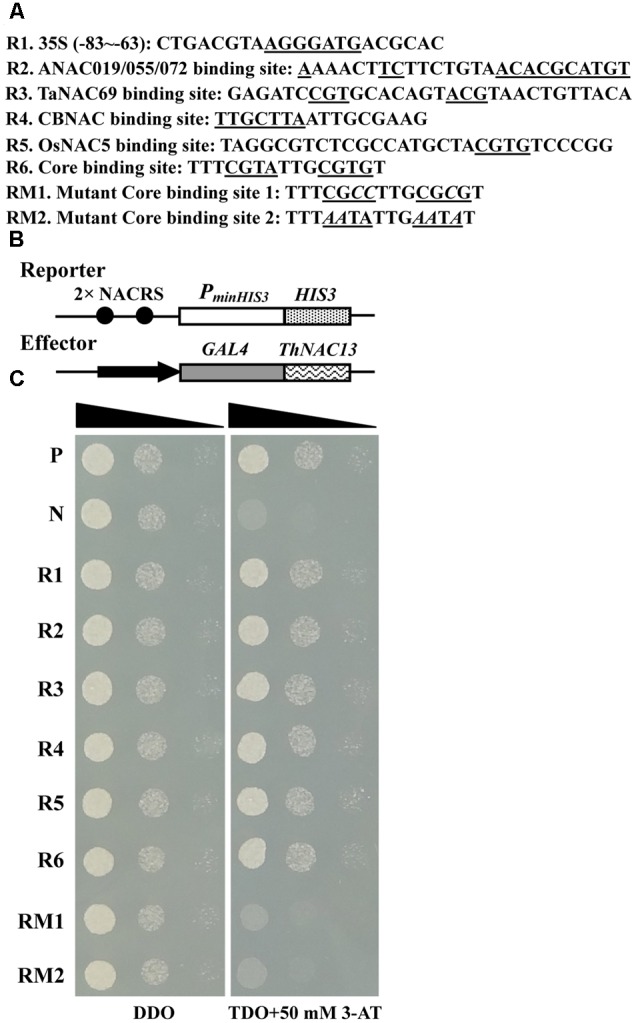
**Identification of the NACRS and CBNACRS elements recognized by ThNAC13. (A)** Oligonucleotides used in construction of the recombinant report vectors. **(B)** Scheme of the reporter and effector vector. **(C)** Analysis of the interactions between the ThNAC13 and NACRS/CBNACRS elements using the yeast one-hybrid. The reporter and effector constructs were co-transformed into the yeast strain Y187. The transformants were further identified by spotting serial dilutions (1, 10^-1^ and 10^-2^) of yeast onto SD/-Leu/-Trp (DDO) and SD/-His/-Leu/-Trp (TDO) plates containing 3-AT (3-amino-1,2,4-triazole). P, positive control (pGAD-Rec2-53 + p53HIS2); N, negative control (pGAD-ThNAC13 + p53HIS2); R1–R6 and RM1–RM2, The positive transformants containing pGAD-ThNAC13 and pHIS2-NACRS/CBNACRS.

To investigate whether ThNAC13 protein can bind to the NACRS and CBNACBS, two tandem copies of these motifs or their mutants were tested for interaction with ThNAC13 using a Y1H system. The sequences for construction of the recombinant report vectors are shown in **Figure 3A**, and the scheme of the reporter and effector vectors used in Y1H are shown in **Figure 3B**. On the SD/-Leu-/Trp medium, all transformants grew well (left panel). On the SD/-Leu-/Trp-/His medium containing 50 mM 3-AT, yeast cells carrying pGAD-ThNAC13/pHIS2-NACRS (R1–R6) grew well, while the mutants RM1 and RM2 and the negative control could not grow (**Figure 3C**). These results indicate that ThNAC13 can bind to both NACRS and CBNACBS but failed to bind to the mutants, which further suggest that ThNAC13 can specifically bind to the NACRS and CBNACBS motifs.

### *ThNAC13* Increases the Tolerance to Salt and Osmotic Stresses

**FIGURE 4:**
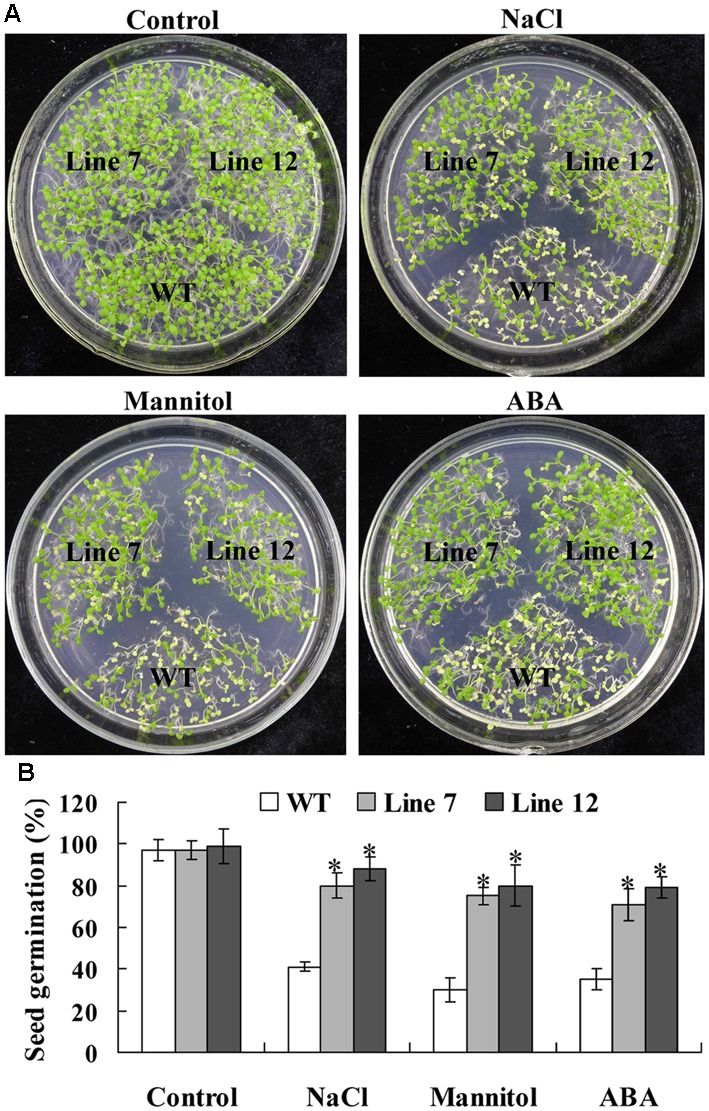
**Comparison of the germination rate between *ThNAC13*-transformed and WT *Arabidopsis* plants. (A)** Seed germination assay between *ThNAC13*-transformed and WT *Arabidopsis* plants. The surface-sterilized seeds were sowed on 1/2 MS solid medium supplemented with 100 mM NaCl, 200 mM mannitol or 2 μM ABA and incubated at 22°C. **(B)** Germination assays of *ThNAC13*-transformed *Arabidopsis* plants. Data are represented as mean ± SD of at least three independent replications. Asterisk indicates significant difference (^∗^*P* < 0.05) between transgenic lines and WT plants.

**FIGURE 5:**
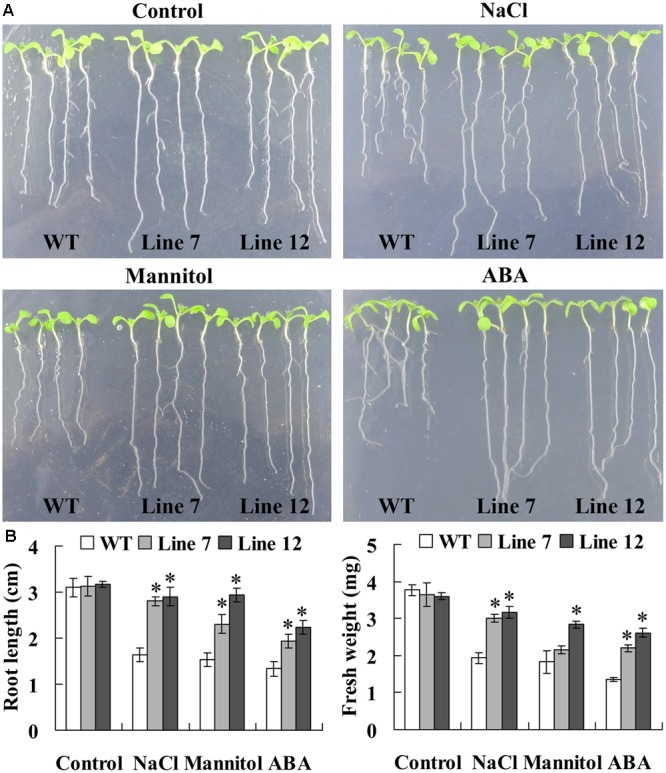
**Comparison of root length and fresh weight between *ThNAC13*-transformed and WT *Arabidopsis* plants. (A)** The growth and phenotype of *ThNAC13*-transformed and WT *Arabidopsis* plants. **(B)** Effects of salt, drought stresses and ABA treatment on root length and fresh weight. Five-day-old seedlings were cultured vertically on 1/2 MS solid medium supplemented with 100 mM NaCl, 200 mM mannitol or 10 μM ABA for 7 days. The photographs were taken, and the root length and fresh weight of each plant were measured. Data are represented as the mean ± SD of at least three independent replications. Asterisk indicates significant difference (^∗^*P* < 0.05) between transgenic lines and WT plants.

Twelve lines of T_3_ homozygous *ThNAC13*-transformed *Arabidopsis* were generated by selection on medium containing kanamycin and further verified by genomic DNA PCR and qRT-PCR (Supplementary Figure S2). Two independent transgenic *Arabidopsis* lines (lines 7 and 12) with high transcript levels of *ThNAC13* were selected for further study. We first evaluated the stress tolerance of transgenic and wild-type (WT) *Arabidopsis* seeds with seed germination tests. There was no difference in germination rates between WT and *ThNAC13*-transformed plants on 1/2 MS medium. However, the seed germination rates of *ThNAC13-*transformed lines were significant higher than those of WT plants under NaCl, mannitol or ABA stress (**Figure 4**), suggesting that overexpression of *ThNAC13* improves salt and osmotic tolerances during the germination stage. Next, 5-day-old *Arabidopsis* seedlings were grown vertically on 1/2 MS solid medium supplemented with or without NaCl, mannitol or ABA. After growth for 7 days, the phenotype was similar between *ThNAC13*-transfomed and WT plants under normal conditions (**Figure 5**), suggesting that overexpression of *ThNAC13* did not influence the growth and phenotype of transgenic plants. Under NaCl, mannitol or ABA stress conditions, the root length and fresh weight gain were significantly increased in *ThNAC13*-transformed plants compared with WT plants, indicating that *ThNAC13* improves the tolerances to NaCl, mannitol or ABA stress.

### Generation of the Transgenic *T*. *hispida* Plants with Overexpression and RNAi-Silencing of *ThNAC13*

For further investigation of the function of *ThNAC13* using gain- and loss-of-function methods, *T. hispida* plants transiently overexpressing *ThNAC13* (OE), RNAi-silenced for *ThNAC13* (IE) and controls (transformed with empty pROKII, VC) were generated. The expression of *ThNAC13* in these 3 transformed *T*. *hispida* plant under different stresses was studied using qRT-PCR. The transcript levels of *ThNAC13* were significantly increased in OE plants and substantially decreased in IE plants compared with control (VC) plants under all the conditions, especially with salt, mannitol or ABA stress for 24 h (Supplementary Figure S3). These results indicate that the genetically transformed *T*. *hispida* plants are suitable for gain- and loss-of-function studies.

### *ThNAC13* Mediates ROS Scavenging Capability

**FIGURE 6:**
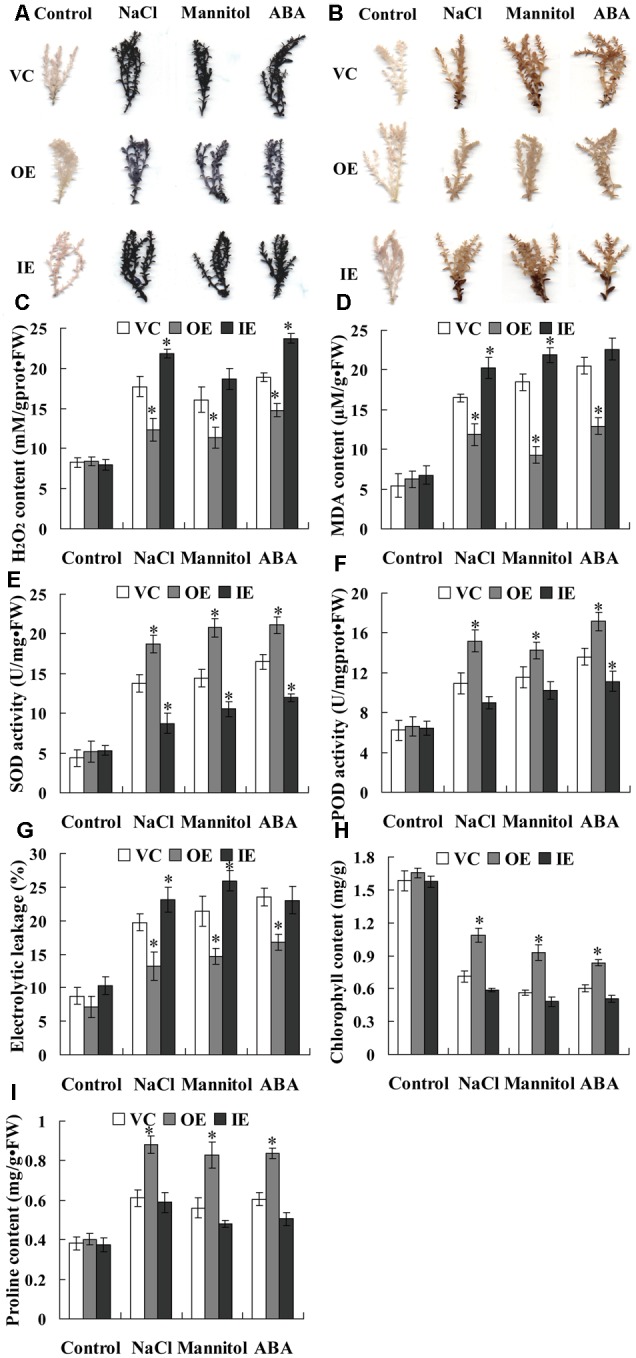
**Histochemical staining and physiological analyses of *ThNAC13*-transformed and WT *Arabidopsis* plants.** Rosette leaves from *ThNAC13*-transformed and WT *Arabidopsis* plants untreated or treated with 150 mM NaCl, 200 mM mannitol or 30 μM ABA for 2 h were used for **(A)** NBT and **(B)** DAB staining. **(C–I)** Physiological analyses among the *Arabidopsis* plants. Four-week-old seedlings of *ThNAC13*-transformed and WT *Arabidopsis* plants were irrigated with 100 mM NaCl, 200 mM mannitol or 30 μM ABA for 2 h (for H_2_O_2_ measurement) and for 48 h (to measure MDA content, SOD and POD activities, electrolyte leakage rate, chlorophyll and proline contents). Data are represented as the mean ± SD of at least three independent replications. Asterisk indicates significant difference (^∗^*P* < 0.05) between transgenic lines and WT plants.

**FIGURE 7:**
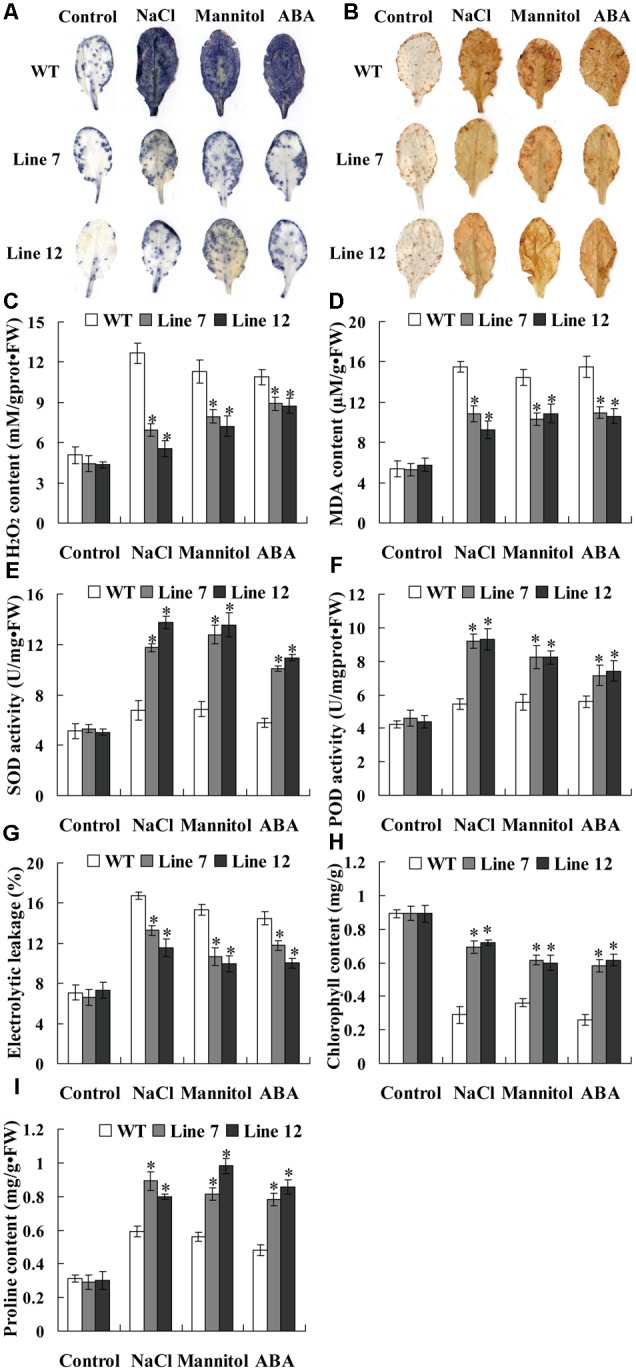
**Histochemical staining and physiological analyses in transgenic *T. hispida* plants. (A)** NBT staining for detecting $O_{2}^{–}$; **(B)** DAB staining for detecting H_2_O_2_. **(C–I)** Physiological analyses among the different transgenic *T. hispida* plants. **(C)** H_2_O_2_ contents; **(D)** MDA contents; **(E)** SOD activity; **(F)** POD activity; **(G)** electrolyte leakage; **(H)** chlorophyll contents; **(I)** proline contents. The *T*. *hispida* plants were grown on 1/2 MS medium or 1/2 MS medium containing 150 mM NaCl, 200 mM mannitol or 30 μM ABA for 24 h and used for analysis. VC, the empty pROKII-transformed *T. hispida* plants; OE, transiently overexpressing *ThNAC13* in *T. hispida* plants; IE, *ThNAC13* RNAi-silenced *T. hispida* plants transiently. Data are represented as the mean ± SD of at least three independent replications. Asterisk indicates significant difference (^∗^*P* < 0.05) between transgenic (OE and IE) and control (VC) plants.

Abiotic stress can induce oxidative stress through generation of reactive oxygen species (ROS), and the accumulation of ROS damages the cell membrane by oxidizing proteins, lipids and DNA (Mittler et al., 2004). NBT and DAB staining were first used for detecting the cellular levels of $O_{2}^{•–}$ and H_2_O_2_ in three types of transiently transformed *T. hispida* plants. The results showed that the transgenic *Tamarix* plants overexpressing *ThNAC13* (OE) had the lowest $O_{2}^{•–}$ and H_2_O_2_ accumulation, and the transgenic *Tamarix* plants with knockdown of *ThNAC13* (IE) had the highest $O_{2}^{•–}$ and H_2_O_2_ accumulation, although they had similar $O_{2}^{•–}$ and H_2_O_2_ levels under normal conditions (**Figures 6A,B**). To confirm the results from *T. hispida*, we further determined the ROS accumulation in *Arabidopsis* plants using *in situ* histochemical staining with NBT and DAB. There was no obvious difference in NBT and DAB staining between *ThNAC13*-transformed and WT plants under normal conditions. However, compared with WT plants, the transgenic plants showed substantially reduced $O_{2}^{•–}$ and H_2_O_2_ accumulation under salt, mannitol and ABA conditions (**Figures 7A,B**).

Additionally, we further measured H_2_O_2_ and MDA contents in transgenic *T. hispida* plants. There was no difference in H_2_O_2_ and MDA contents among OE, VC, and IE plants under normal conditions. However, under stress conditions, IE plants had the highest level, followed by IE plants, and OE had the lowest H_2_O_2_ levels (**Figures 6C,D**). Consistent with these results, transgenic *Arabidopsis* plants overexpressing *ThNAC13* displayed low H_2_O_2_ and MDA contents compared with WT plants under stress conditions (**Figures 7C,D**). As ROS levels were decreased by expression of *ThNAC13*, we investigated whether SOD and POD activities were regulated by *ThNAC13*. Among all the studied *Tamarix* plants, there was no significant difference in SOD and POD activities under normal conditions. The SOD and POD activities were also markedly elevated in OE plants and reduced in IE plants compared with VC plants (**Figures 6E,F**). We further studied SOD and POD activities in *Arabidopsis*. There was no significant difference in the activities of SOD and POD between transgenic and WT plants under normal conditions. Compared with WT *Arabidopsis* plants, the activities of POD and SOD in *ThNAC13*-transfomed *Arabidopsis* plants were significantly increased under the different stress conditions (**Figures 7E,F**), which was consistent with the results obtained from *T*. *hispida* plants. Therefore, overexpression of *ThNAC13* significantly activates SOD and POD activities under abiotic stress conditions.

### Analysis of Electrolyte Leakage Rate and the Contents of Chlorophyll and Proline

To estimate the level of abiotic stress damage to the studied plants, several stress-related physiological changes were measured. Under normal conditions, all transgenic *T. hispida* plants displayed similar electrolytic leakage rates, chlorophyll and proline contents (**Figures 6G–I**). However, the electrolytic leakage rates in IE plants were the highest, followed by VE, and were the lowest in OE plants (**Figure 6G**). Meanwhile, OE plants had the highest contents of chlorophyll and proline, followed by VC plants, and IE plants had the lowest contents of chlorophyll and proline (**Figures 6H,I**). The electrolyte leakage rates in transgenic *Arabidopsis* plants overexpressing *ThNAC13* were decreased compared with WT plants under salt, osmotic or ABA stress (**Figure 7G**). Furthermore, the contents of chlorophyll and proline in *ThNAC13*-transformed *Arabidopsis* were both significantly higher than those in WT plants (**Figures 7H,I**). These results are consistent with the results from *T. hispida*, indicating the reliability of these findings.

### *ThNAC13* Induces the Expression of *SOD*s and *POD*s

**FIGURE 8:**
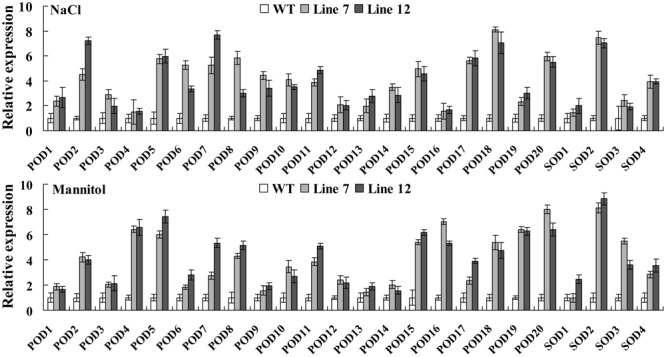
**Analysis of the expression of the *POD* and *SOD* genes in *ThNAC13*-transformed *Arabidopsis* plants.** Four-week-old seedlings of *ThNAC13*-transformed and WT *Arabidopsis* plants were treated with 100 mM NaCl or 200 mM mannitol for 24 h, and were harvested for qRT-PCR analysis. The transcription levels of *SOD*s or *POD*s in WT plants under the same condition were set as a calibrator to 1 to calculate their expression in transgenic plants. The error bars were calculated from three replicates. The *Tair* locus numbers of *Arabidopsis POD*s and *SOD*s are shown in Supplementary Table S1.

As the activities of POD and SOD were both markedly elevated in the *Arabidopsis* plants overexpressing *ThNAC13*, we further analyzed the transcription levels of *POD* and *SOD* genes, which were shown to have enzymatic activities in previous studies. The expression of many studied genes was highly increased in *ThNAC13*-transformed *Arabidopsis* under NaCl and mannitol treatments (**Figure 8**). These results suggested that *ThNAC13* can induce the transcription levels of *SOD* and *POD* genes, which will enhance the activities of SOD and POD.

## Discussion

### ThNAC13 Is a Member of the NAC Transcription Factors

Most NAC proteins share a well-conserved N-terminal DNA binding domain and a diversified C-terminal domain (Puranik et al., 2012; Wang et al., 2014b). In this study, we found that the ThNAC13 protein has the conserved NAC domain at N-terminus. Multiple sequence alignments showed that ThNAC13 has high amino acid sequence identity with the reported stress-responsive NAC proteins of *Arabidopsis* (AtNAC019, AtNAC055, and AtNAC072) (Supplementary Figure S1), suggesting that ThNAC13 may also be a stress-responsive NAC. Previous studies have shown that NAC proteins could bind to the NACRS containg the core sequence “CGT[A/G]” (Duval et al., 2002; Tran et al., 2004; Olsen et al., 2005; Xue, 2005; Takasaki et al., 2010; Hao et al., 2011). However, Kim et al. (2007) found that the *Arabidopsis* transcriptional repressor CBNAC can bind to CBNACBS with a “GCTT” sequence but not the “CGT[A/G]” sequence, indicating that different NAC transcription factors may bind to different element sequences. Our study suggested that ThNAC13 can bind to the NACRS element; at the same time, it can also bind to a motif sequence containing the “GCTT,” similar to CBNAC (**Figure 3C**). These results suggested that ThNAC13 could bind to NACRS and CBNACBS to regulate gene expression.

Transactivation assays showed that the C-terminal of ThNAC13 has a high transactivation ability, although the full-length protein does not have this capacity (**Figure 2B**). However, when the N-terminal of ThNAC13 is present, the transactivation is abolished. These results indicated that the N-terminal of ThNAC13 may have a transcriptional repression domain, which leads to abolished transactivation. Previous studies also support our hypothesis. For example, the conserved hydrophobic LVFY motif was a transcription repression domain that is present in the N-terminal of GmNAC20 protein (Hao et al., 2010). We also found the LVFY motif in N-terminal of ThNAC13 (Supplementary Table S2). However, ThNAC13 could regulate the expression of *POD* and *SOD* genes (**Figure 8**). This is probably due to its C-terminal domain, which has high transcriptional activation activity, or the interplay among the C-terminal activation domain, the DNA-binding domain and NARD (NAC Repression Domain). Therefore, ThNAC13 may act as a transcriptional repressor or activator, depending on interactions with other transcription factors or conformational changes.

### *ThNAC13* Improves Tolerance to Salt, Osmotic, and ABA Stresses

Recently, numerous reports have shown that NAC transcription factors play important roles in the response to different abiotic stresses and function as positive stress response TFs (Jeong et al., 2013; Mao et al., 2014; Fang et al., 2015). In the present study, our results showed that *ThNAC13* can induce a series of physiological changes to improve tolerance to salt, osmotic and ABA stresses (**Figures 4, 6, 7**). These results suggested that ThNAC13 functions as a stress-responsive protein and enhances the salt and osmotic tolerance. In addition, *ThNAC13* promotes tolerance to ABA, and NADPH oxidases are involved in abiotic stress tolerance and regulated by ABA, which plays an important role in ABA-mediated stomatal closure (Acharya et al., 2013). Therefore, there may be a relationship between ThNAC13 and NADPH oxidase activity, which deserves further study.

### ThNAC13 Improves ROS-Scavenging Capability

Plants are commonly exposed to various adverse environments, which result in accumulation of ROS, damage to protein synthesis and stability, cellular macromolecules and membrane lipids in plant cells and generation of oxidative stress (Wang et al., 2005). Therefore, ROS-scavenging is important for plant tolerance. Two prominent ROS species, $O_{2}^{•–}$ and H_2_O_2_, are considered to be important signaling molecules in plant cells. In this study, biochemical staining (NBT and DAB) showed that the ROS accumulation ($O_{2}^{•–}$ and H_2_O_2_) in transgenic *Tamarix* and *Arabidopsis* plants overexpressing *ThNAC13* was lower than that in control (VC or WT) plants under abiotic stress and ABA treatment (**Figures 6A,B, 7A,B**). MDA, the product of lipid peroxidation caused by ROS, is also used to evaluate ROS-mediated injuries in plants (Moore and Roberts, 1998). We found that the H_2_O_2_ and MDA contents were both decreased by expression of *ThNAC13* under abiotic stress and ABA treatment (**Figures 6C,D, 7C,D**). These results suggested that ThNAC13 can reduce ROS accumulation and decrease lipid peroxidation to improve tolerance to salt and osmotic stresses.

The antioxidant enzymes, SOD and POD, are known to play important roles in ROS scavenging, which influences the cellular ROS levels. We found that the activities of SOD and POD were significantly induced by *ThNAC13* under different stress conditions. Furthermore, most of the studied *SOD* and *POD* genes were activated in *ThNAC13* transgenic *Arabidopsis* plants, which may lead to increased SOD and POD activities in plants overexpressing *ThNAC13* (**Figures 6E,F, 7E,F, 8**). These results suggest that *ThNAC13* improves stress tolerance at least partly through enhancing ROS-scavenging capability, which will decrease the degree of membrane lipid peroxidation. In addition, we found that the core sequences of NACRS and CBNACBS elements were both enriched in the promoters (<1.5 kb) of the studied *SOD*s and *POD*s (Supplementary Table S3), suggesting that ThNAC13 may bind to these elements to directly regulate the expression of these genes. These results suggest that *ThNAC13* improves tolerance to salt and osmotic stresses by regulating the transcript levels of *SOD*s and *POD*s to increase the ROS-scavenging capability.

### *ThNAC13* Induces Proline Biosynthesis

Plant proline could protect cells by serving as an osmotic agent and also as a radical scavenger under biotic or abiotic stress. In addition, when stress is relieved, the accumulated proline could be degraded as a supply of energy for plant growth (Kavi Kishor and Sreenivasulu, 2014). In this study, proline content was positively correlated with the transcripts of *ThNAC13* (**Figure 6I**), and *Arabidopsis* plants overexpressing *ThNAC13* displayed increased proline contents under different stresses (**Figure 7I**), indicating that ThNAC13 induces the biosynthesis of proline, and the increased proline enhances both osmotic potential and ROS-scavenging capability to improve abiotic stress tolerance.

## Conclusion

A stress-responsive *NAC* gene from *T*. *hispida, ThNAC13*, was identified and functionally characterized. ThNAC13 functions as a transcription factor that improves salt and osmotic tolerance by increasing ROS-scavenging capability and osmotic potential. This study improves our understanding of the biological functions of NAC proteins in response to abiotic stress in woody plants.

## Author Contributions

LW and ZL carried out all the experiments and data analysis. LW, ZL, and YW conceived the project, designed the experiments and drafted the manuscript. ML and YW supervised the analysis and critically revised the manuscript. All authors read and approved the final manuscript.

## Conflict of Interest Statement

The authors declare that the research was conducted in the absence of any commercial or financial relationships that could be construed as a potential conflict of interest.

## Footnotes

1 http://meme-suite.org
