# Supplementary material for: ThNAC13, a NAC Transcription Factor from Tamarix hispida, Confers Salt and Osmotic Stress Tolerance to Transgenic Tamarix and Arabidopsis
Source: Front Plant Sci. 2017 Apr 26;8:635. doi: 10.3389/fpls.2017.00635 (PMC5405116; doi:10.3389/fpls.2017.00635)
Supplement: Supplementary file 1 [file Table_1.DOC]

**TABLE S1 Primers used in this study.**

| **Primers used for real-time RT-PCR** | | | |
| --- | --- | --- | --- |
| Genes | GenBank number | Forward and reverse primers (5’–3’) | |
| *Tamarix hispida* | | | |
| *ThNAC13* | JQ974967 | CAACGAGGAACGGTAGCAG | TCGATGTGGGAGGAGGAAG |
| *β-actin* | FJ618517 | AAACAATGGCTGATGCTG | ACAATACCGTGCTCAATAGG |
| *α-tubulin* | FJ618518 | CACCCACCGTTGTTCCAG | ACCGTCGTCATCTTCACC |
| *β-tubulin* | FJ618519 | GGAAGCCATAGAAAGACC | CAACAAATGTGGGATGCT |
| *Arabidopsis thaliana* | | | |
| *POD1* | AT1G05260 | CTTTCACAAACCGTCTCTAC | AGTGGTGAGAGCAGAGTCTG |
| *POD2* | AT1G14550 | CCATAGGACAATCTCAATGC | TGATCGGTTACTAATAGTC |
| *POD3* | AT1G24110 | TCTGACCGTTCAAGAAATGG | TGGAGCAACCCGTAACCGTG |
| *POD4* | AT1G30870 | TGTGGCACCATCCAGTCGAG | CTGCGAAAGTCTTTACAAGC |
| *POD5* | AT1G65970 | CAGTATGAGCCATGTGCCTG | CAAGCAACAAAGCGAATCTC |
| *POD6* | AT2G18140 | TCCGGGAGCCACACCATTGG | TGGTCGGAATTCAACAGTC |
| *POD7* | AT2G18150 | CCAATCCGGAAACGGAAGTC | TCTGCATACTTCTTGACGAG |
| *POD8* | AT3G49110 | GCAACACTGGATTACCTGAC | CCATCAGCATATGCTCTCAC |
| *POD9* | AT3G50990 | AGGTTATACAACCATACTGG | CGTAATACTTGACCATCTC |
| *POD10* | AT4G11290 | TCGACAGCGAATATGCCGAC | GAACTCTTGCTCCGATCCTC |
| *POD11* | AT4G17690 | GAATGGTTTCACTCTAAAGG | GGAAGCTAACAGTCCAAGAC |
| *POD12* | AT4G25980 | GAACAACGGCCTGCTTCTTC | TCCACGACCTGTCTGGTCG |
| *POD13* | AT4G26010 | TCCAGGACAGGCTTTCCGAC | GAAGAGTGTATTGCTTGATG |
| *POD14* | AT4G30170 | AGCCGTCACGGCCTCTCTC | CAAGATTTGATCTGACGTG |
| *POD15* | AT5G47000 | GACTGTTCCTGACATCCAC | CTTGAAGTACATGTTGTCG |
| *POD16* | AT5G51890 | CTTGTCGGTGAAAGACATG | GACCCAAACACTCCTTTCC |
| *POD17* | AT5G58390 | ATCCCTCCTCCGATCACTAC | GTCGAACCTATCGGGAGAG |
| *POD18* | AT5G58400 | GGCAAGCCAGGTGCGTCAC | TCCGGCTGTAGGATACGAC |
| *POD19* | AT5G66390 | CTCACTAAGTTCAAGCGTC | GAATAGGGTCTGGTCACCTC |
| *POD20* | AT5G64110 | CTGGACATACGATAGGAACG | GACTCGAGGAGACCTCGAC |
| *SOD1* | AT1G12520 | GTCACCCGGAACCCACAGC | CCGAATAAAAGGCCTCTCC |
| *SOD2* | AT3G56350 | GAAGGAGGTGGCAAACCAC | TCTTGTACTGTGGATAGTAG |
| *SOD3* | AT5G23310 | CGCTGCACAGGTCTATAACC | AATATCGTCCCACACGAGTG |
| *SOD4* | AT5G51100 | CCTGGAGGTGGAGGAAAGC | CTGCATTGGGCGTCTTCAC |
| *α-tubulin* | At1G50010 | GATGTACCGTGGTGATGTC | GAGCCTCTGAAAATTCTCC |
| *Ubiquitin* | AT1G55060 | GGAAAGCAGCTCGAAGATG | AAGCTTCCACCGCGGAGAC |

| Constructs | Forward and reverse primers (5’–3’) | |
| --- | --- | --- |
| **Primers used in subcellular localization analysis** | | |
| pBI121-ThNAC13-GFP | TCTAGACTGGTACCCGGGATGGGTTTGGCAGAGAGAGATC | CTAGTCAGTCGACCCGGGTACCGCGTGAACCCGCACCCA |
| pBI121-GFP | TTTCATTTGGAGAGAACACG | CGACCAGGATGGGCACCAC |
| **Primers used in constructing yeast recombinant plasmid** | | |
| pBD-ThNAC13_1-373_ (2) | CATGGAGGCCGAATTCATGGGTTTGGCAGAGAGAG | GCAGGTCGACGGATCCTCACCGCGTGAACCCGCAC |
| pBD-ThNAC13_1-165_ (3) | CATGGAGGCCGAATTCATGGGTTTGGCAGAGAGAG | GCAGGTCGACGGATCCTTTCTTGTATATCCTACAT |
| pBD-ThNAC13_166-373_ (4) | CATGGAGGCCGAATTCAGCTCAGCGTCATCAAC | GCAGGTCGACGGATCCTCACCGCGTGAACCCGCAC |
| pBD-ThNAC13_166-300_ (5) | CATGGAGGCCGAATTCAGCTCAGCGTCATCAAC | GCAGGTCGACGGATCCCTGAACCGCAGATTGCTGC |
| pBD-ThNAC13_166-280_ (6) | CATGGAGGCCGAATTCAGCTCAGCGTCATCAAC | GCAGGTCGACGGATCCCGTCGGGAAGTCGTCGGT |
| pBD-ThNAC13_166-252_ (7) | CATGGAGGCCGAATTCAGCTCAGCGTCATCAAC | GCAGGTCGACGGATCCACCGGACCCCAATCGCAAG |
| pBD-ThNAC13_166-226_ (8) | CATGGAGGCCGAATTCAGCTCAGCGTCATCAAC | GCAGGTCGACGGATCCCGGGCGGTTGATTTCC |
| pBD-ThNAC13_227-373_ (9) | CATGGAGGCCGAATTCTTCCCTGCCGATCTCCCT | GCAGGTCGACGGATCCTCACCGCGTGAACCCGCAC |
| pBD-ThNAC13_227-353_ (10) | CATGGAGGCCGAATTCTTCCCTGCCGATCTCCCT | GCAGGTCGACGGATCCCGTGGTGGGAAAGCCCTG |
| pBD-ThNAC13_227-330_ (11) | CATGGAGGCCGAATTCTTCCCTGCCGATCTCCCT | GCAGGTCGACGGATCCAGCTAGCTGAGTATTCC |
| pBD-ThNAC13_227-300_ (12) | CATGGAGGCCGAATTCTTCCCTGCCGATCTCCCT | GCAGGTCGACGGATCCCTGAACCGCAGATTGCTGC |
| pBD-ThNAC13_253-373_ (13) | CATGGAGGCCGAATTCAATTTTGACTGGACCGCG | GCAGGTCGACGGATCCTCACCGCGTGAACCCGCAC |
| pBD-ThNAC13_281-373_ (14) | CATGGAGGCCGAATTCGCTTATACGCAAGGAGCAAC | GCAGGTCGACGGATCCTCACCGCGTGAACCCGCAC |
| pBD | TCATCGGAAGAGAGTAGT | TTCGTTTTAAAACCTAAGAGTC |
| **Primers used in NARS motif binding analysis** | | |
| pGAD-ThNAC13 | TGGCCATTATGGCCCGGGATGGGTTTGGCAGAGAGAGATC | GACATGTTTTTTCCCGGGTCACCGCGTGAACCCGCAC |
| pAD-Rec2 | ATGAACATGGAGGCCAGTG | GATGGATCCCGTATCGATG |
| R1 (CaMV 35S (-83~-63)) | AATTC(CTGACGTA**AGGGATG**ACGCAC)_2_GAGCT | C(GTGCGT**CATCCCT**TACGTCAG)_2_G |
| R2 (ANAC019/055/072 binding site) | AATTC(**A**AAACT**TC**TTCTGTA**ACACGCATGT**)_2_GAGCT | C(**ACATGCGTGT**TACA**GA**AGAAGTTT**T**)_2_G |
| R3 (TaNAC69 binding site) | AATTC(GAGATC**CGT**GCACAGT**ACG**TAACTGTTACA)_2_GAGCT | C(TGTAACAGTTA**CGT**ACTGTGC**ACG**GATCTC)_2_G |
| R4 (CBNAC binding site) | AATTC(**TTGCTTA**ATTGCG**AAG**)_2_GAGCT | C(**CTT**CGCAAT**TAAGCAA**)_2_G |
| R5 (OsNAC5/6 binding site) | AATTC(TAGGCGTCTCGCCATGCTA**CGTG**TCCCGG)_2_GAGCT | C(CCGGGA**CACG**TAGCATGGCGAGACGCCTA)_2_G |
| R6 (Core binding site) | AATTC(TTT**CGTA**TTG**CGTG**T)_2_GAGCT | C(A**CACG**CAA**TACG**AAA)_2_G |
| RM1 (Mutant Core binding site 1) | AATTC(TTT**CG*CC***TTG**CG*C*G**T)_2_GAGCT | C(A**C*G*CG**CAA***GG*CG**AAA)_2_G |
| RM2 (Mutant Core binding site 2) | AATTC(TTT***AA*TA**TTG***AA*T*A***T)_2_GAGCT | C(A***T*A*TT***CAA**TA*TT***AAA)_2_G |
| **Primers used in constructing plant plasmids** | | |
| pROKII-ThNAC13 | CTCTAGAGGATCCCCGGGATGGGTTTGGCAGAGAGAGATC | TCGAGCTCGGTACCCGGGTCACCGCGTGAACCCGCAC |
| pROKII | TTTCATTTGGAGAGAACACG | TGCCAAATGTTTGAACGATC |
| pFGC5941-ThNAC13 | ThNAC13-Sense-F: TTGGCGCGCCCCTGCCGATCTCCCTTGCGTG | ThNAC13-Sense-R: CATGATTTAAATGAAGCGGCGGCAGTGAAAG |
|  | ThNAC13-Anti-F: CCTTAATTAACCTGCCGATCTCCCTTGCGTG | ThNAC13-Anti-R: TGCTCTAGAGAAGCGGCGGCAGTGAAAG |
