# Supplementary material for: ThNAC13, a NAC Transcription Factor from Tamarix hispida, Confers Salt and Osmotic Stress Tolerance to Transgenic Tamarix and Arabidopsis
Source: Front Plant Sci. 2017 Apr 26;8:635. doi: 10.3389/fpls.2017.00635 (PMC5405116; doi:10.3389/fpls.2017.00635)
Supplement: Supplementary file 2 [file Table_2.DOC]

**TABLE S2 Distribution of conserved motifs in the NAC proteins.**

| **Motif no.** | **E-value** | **Sites** | **Width (aa)** | **Annotation of motif** | **Conserved amino acids of motif** |
| --- | --- | --- | --- | --- | --- |
| 1 | 2.4e-733 | 15 | 70 | NAC Subdomain | FRFHPTDEELVVHYLCRKCAGHPFSVPIIAEIDLYKFDPWDLPSKALFGEKEWYFFSPRDRKYPNGSRPN |
| 2 | 1.1e-358 | 15 | 39 | NAC Subdomain | GRPVGIKKALVFYAGKAPKGVKTNWIMHEYRLADVDRSA |
| 3 | 3.1e-156 | 15 | 22 | NAC Subdomain | NSLRLDDWVLCRIYKKKGSAEK |
| 4 | 4.7e-096 | 10 | 20 | NAC Subdomain | RVAGSGYWKATGTDKIITTE |
| 5 | 7.7e-027 | 2 | 70 | - | EQKPVVAGPAFPDLAAYYDRPSDSMPRLHADSSCSEQVLSPEFACEVQSQPKISEWERTFATVGPINPAA |
| 6 | 2.4e-023 | 6 | 26 | - | CSNGSSSSSSSHLDDVLESLPEIDDR |
| 7 | 1.0e-014 | 4 | 20 | - | GGGGSDPLLQDILMYWGKPF |
| 8 | 4.3e-004 | 2 | 28 | - | AAAVAAAGMVSSGGGVQRKPMVGVNAAV |
| 9 | 9.4e-003 | 6 | 35 | - | QQQQQEEKTGFPGLAEGXXFDWANXSGLNSVSESV |
| 10 | 2.2e-002 | 2 | 37 | - | LCHVDTMVDAKLEEEVQSGVRNQRVDGNGHFLPNDDT |

*The motif numbers correspond to the numbers in **FIGURE 1**.
