# Supplementary material for: ThNAC13, a NAC Transcription Factor from Tamarix hispida, Confers Salt and Osmotic Stress Tolerance to Transgenic Tamarix and Arabidopsis
Source: Front Plant Sci. 2017 Apr 26;8:635. doi: 10.3389/fpls.2017.00635 (PMC5405116; doi:10.3389/fpls.2017.00635)
Supplement: Supplementary file 3 [file Table_3.DOC]

**TABLE S3 Distribution of NACRS and CBNACBS elements in the promoters of *POD* and *SOD* genes.**

| **Genes** | **Locus tag** | **< 1.5 Kb promoter region** | |
| --- | --- | --- | --- |
|  |  | **NACRS number** | **CBNACBS number** |
| *POD1* | AT1G05260 | 11 | 3 |
| *POD2* | AT1G14550 | 14 | 4 |
| *POD3* | AT1G24110 | 3 | 2 |
| *POD4* | AT1G30870 | 13 | 7 |
| *POD5* | AT1G65970 | 9 | 11 |
| *POD6* | AT2G18140 | 9 | 2 |
| *POD7* | AT2G18150 | 7 | 9 |
| *POD8* | AT3G49110 | 14 | 6 |
| *POD9* | AT3G50990 | 6 | 2 |
| *POD10* | AT4G11290 | 6 | 11 |
| *POD11* | AT4G17690 | 7 | 13 |
| *POD12* | AT4G25980 | 2 | 2 |
| *POD13* | AT4G26010 | 3 | 1 |
| *POD14* | AT4G30170 | 7 | 6 |
| *POD15* | AT5G47000 | 13 | 6 |
| *POD16* | AT5G51890 | 13 | 9 |
| *POD17* | AT5G58390 | 10 | 6 |
| *POD18* | AT5G58400 | 18 | 11 |
| *POD19* | AT5G66390 | 9 | 8 |
| *POD20* | AT5G64110 | 12 | 7 |
| *SOD1* | AT1G12520 | 0 | 2 |
| *SOD2* | AT3G56350 | 17 | 11 |
| *SOD3* | AT5G23310 | 2 | 6 |
| *SOD4* | AT5G51100 | 4 | 7 |
