# Supplementary material for: ThNAC13, a NAC Transcription Factor from Tamarix hispida, Confers Salt and Osmotic Stress Tolerance to Transgenic Tamarix and Arabidopsis
Source: Front Plant Sci. 2017 Apr 26;8:635. doi: 10.3389/fpls.2017.00635 (PMC5405116; doi:10.3389/fpls.2017.00635)
Supplement: Supplementary file 4 [file Image_1.PDF]

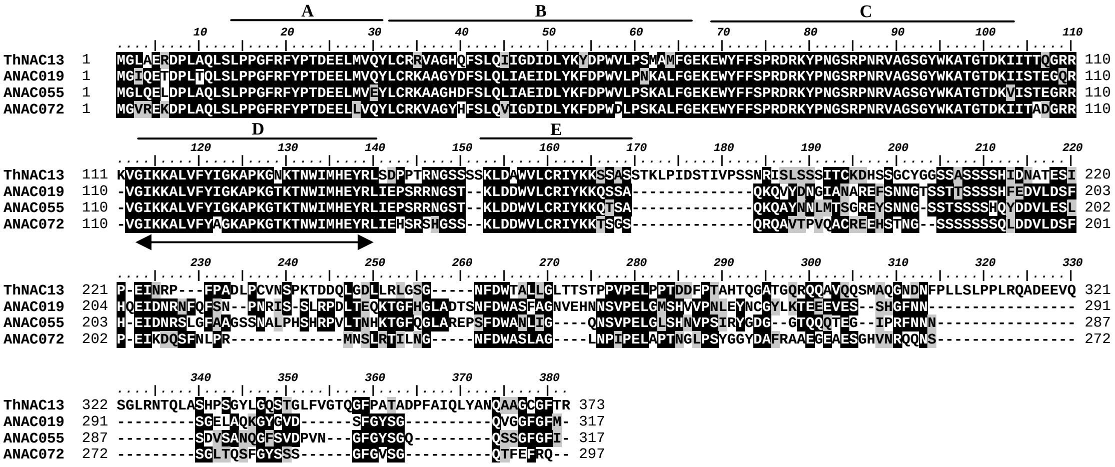

FIGURE S1 Identification of the conserved NAC sub-domains and the putative nuclear localization signal in ThNAC13 protein. Multiple sequence alignments of ThNAC13 and three representative *Arabidopsis* NACs were performed with ClustalW using BioEdit software. The consensus NAC subdomains (A–E) are indicated by lines above the sequences. The putative nuclear localization signal is shown by a double-headed arrow below the sequence.
