# Supplementary material for: ThNAC13, a NAC Transcription Factor from Tamarix hispida, Confers Salt and Osmotic Stress Tolerance to Transgenic Tamarix and Arabidopsis
Source: Front Plant Sci. 2017 Apr 26;8:635. doi: 10.3389/fpls.2017.00635 (PMC5405116; doi:10.3389/fpls.2017.00635)
Supplement: Supplementary file 5 [file Image_2.PDF]

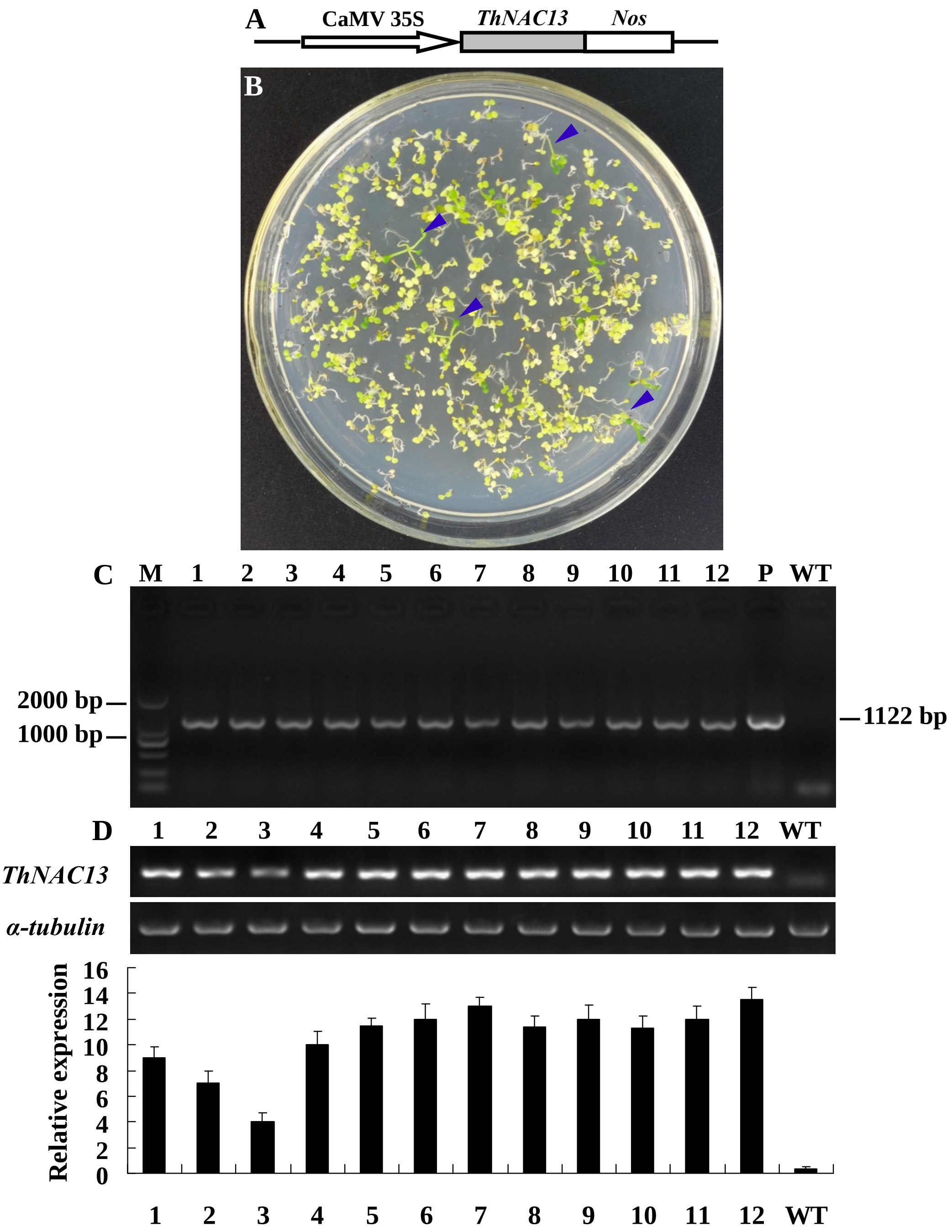


**FIGURE S2 Selection and PCR analysis of *ThNAC13*-transformed *Arabidopsis*.** **(A)** Schematic map of the recombinant plasmid pROKII-ThNAC13; **(B)** Resistance screening of *ThNAC13*-transformed *Arabidopsis*. **(C)** PCR analysis of *ThNAC13*-transformed *Arabidopsis* using genome DNA as PCR template. **(D)** qRT-PCR results showing the *ThNAC13* expression in the 12 independent lines of *ThNAC13* transgenic and WT *Arabidopsis* plants. Parallel reactions using *α-tubulin* (AT1G50010, as an internal control) in primers were carried out to normalize the

amounts of added template. M: DNA Marker DL2000; 1–12: *ThNAC13*-transformed *Arabidopsis* plant line; P: The recombinant plasmid pROKII-ThNAC13; WT: Wild-type.
