# Supplementary material for: ThNAC13, a NAC Transcription Factor from Tamarix hispida, Confers Salt and Osmotic Stress Tolerance to Transgenic Tamarix and Arabidopsis
Source: Front Plant Sci. 2017 Apr 26;8:635. doi: 10.3389/fpls.2017.00635 (PMC5405116; doi:10.3389/fpls.2017.00635)
Supplement: Supplementary file 6 [file Image_3.PDF]

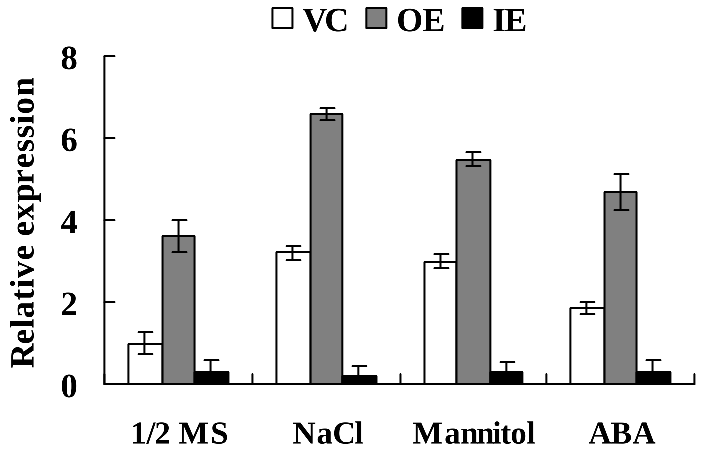


**FIGURE S3 Analysis of the expression of *ThNAC13* in the different kinds of transgenic *T. hispida* plants.** The expression of *ThNAC13* was determined under normal growth conditions and treatment with 150 mM NaCl, 200 mM mannitol or 30 μM ABA for 24 h. The expression level of *ThNAC13* in control plants under normal growth conditions was used as the calibrator (designed as 1). VC: the pROKII vector control transformed *T. hispida* plants; OE: overexpressing of *ThNAC13* in *T. hispida* plants; IE: *ThNAC13* RNAi-silenced *T. hispida* plants. The error bars were standard deviations, which calculated from multiple replicates of the qRT-PCR.
